# Research Progress Regarding Psychrotrophic *Pseudomonas* in Aquatic Products: Psychrophilic Characteristics, Spoilage Mechanisms, Detection Methods, and Control Strategies

**DOI:** 10.3390/foods14030363

**Published:** 2025-01-23

**Authors:** Jingjing Wang, Jing Xie, Jun Mei

**Affiliations:** 1 College of Food Science and Technology, Shanghai Ocean University, Shanghai 201306, China; m230351079@st.shou.edu.cn; 2 National Experimental Teaching Demonstration Center for Food Science and Engineering, Shanghai Ocean University, Shanghai 201306, China; 3 Shanghai Engineering Research Center of Aquatic Product Processing and Preservation, Shanghai 201306, China; 4 Key Laboratory of Aquatic Products High-Quality Utilization, Storage and Transportation (Co-Construction by Ministry and Province), Ministry of Agriculture and Rural Affairs, Shanghai 201306, China

**Keywords:** aquatic products, *Pseudomonos* spp., biofilms, spoilage metabolite, cold-adapted mechanism, control techniques

## Abstract

Aquatic products are an important part of the human diet, but they are easily contaminated by *Pseudomonas* spp., which leads to food deterioration and economic loss. In this paper, the main characteristics of psychrotrophic *Pseudomonas* in aquatic products are reviewed, including its growth adaptation mechanism and biofilm formation ability at low temperatures, and the key role of psychrotrophic *Pseudomonas* in aquatic product spoilage is emphasized. Studies have shown that psychrotrophic *Pseudomonas* can produce a variety of volatile compounds by decomposing proteins and amino acids, affecting the sensory quality and safety of aquatic products. A variety of control strategies to extend the shelf life of aquatic products have been explored, including physical, chemical, and biological methods, particularly biofilm-specific inhibition techniques such as inhibition of quorum sensing and the application of natural antimicrobials. Future research should prioritize the development of novel anti-biofilm products to address the growing problem of psychrotrophic *Pseudomonas* contamination in the aquatic product industry to ensure food safety and public health.

## 1. Introduction

Aquatic products such as fish, shrimp, crab, and shellfish constitute a significant component of the human daily diet (Table 1). They offer a diverse array of vital nutrients, delivering high-quality proteins while maintaining a low fat content [1]. Nonetheless, aquatic products serve as a significant nutrient source for pathogenic foodborne microorganisms, which proliferate easily, leading to heightened food waste and economic losses for both the aquatic products industry and consumers [2]. The high-quality transportation of aquatic products has always been a problem to be solved. The transportation of aquatic products typically involves low-temperature conditions. Low-temperature conditions can inhibit microorganism growth, reduce enzyme activity, and slow down the deterioration of fresh food [3]. Nevertheless, the cold chain also has limitations, as bacteria still active at low temperatures promote microbiological degradation of food, in particular *Pseudomonas* spp. [4]. For instance, some *Pseudomonas* spp. species have evolved to low temperatures, such as *P. syringae* ESC-10 [5], which is able to live 30 days at 2 °C [6].

Microorganisms, especially hardy bacteria, are an important factor in the spoilage of aquatic products. They can be divided into indigenous and non-indigenous bacteria, among which indigenous bacteria include *Pseudomonas* spp., *Escherichia coli*, *Microbacillus*, etc., while non-indigenous bacteria include *Staphylococcus aureus*, *Salmonella*, *Vibrio parahaemolyticus* and so on. *Pseudomonas* spp. are the predominant microorganisms linked to spoilage [11,12]. These microorganisms are linked to spoilage, exhibiting symptoms such as mucosity, altered food texture, unpleasant odors, and off-flavors, which reduce the quality of food products and result in decreased consumer acceptance [13]. *Pseudomonas* spp. in aquatic products possess the capacity to generate biofilms, which can increase food contamination, characterized by complex community structures resulting from the aggregation of extracellular secretions after bacterial adhesion to surfaces. Biofilms function as protective mechanisms, allowing bacteria to endure harsh environmental conditions [14,15]. Biofilms enhance the resistance of spoilage bacteria and expedite the deterioration of aquatic items [16]. *Pseudomonas* spp. decomposes amino acids and proteins in aquatic products, yielding numerous spoilage metabolites, including volatile sulfides and their derivatives, biogenic amines (such as putrescine, cadaverine, and histamine), alcohols, aldehydes, ketones, phenols, and organic acids with offensive odors [17]. Therefore, it is vital to regulate the synthesis of spoilage metabolites in aquatic goods to maintain the quality of aquatic products and food safety.

This review presents a description of the main characteristics of *Pseudomonas* spp. as the main spoilage microorganism in aquatic products, especially their cold-tolerant properties, and reports the latest research progress in *Pseudomonas* spp. detection methods. It further explores various strategies for preventing *Pseudomonas* contamination in the aquatic products industry, offering a theoretical foundation for extending the shelf life of these products.

## 2. Characteristics of *Pseudomonas* spp.

### 2.1. Species of Pseudomonas spp. in Different Aquatic Products

*Pseudomonas* is a genus of Gram-negative, rod-shaped, non-spore-forming, obligate aerobic bacteria, representing the most prevalent genus of Gram-negative bacteria [18]. Because of their versatile metabolic capacity and great adaptability, they are ubiquitous in aquatic and terrestrial ecosystems, and they are one of the bacteria that show excellent adaptability to a variety of environmental conditions [19]. Most *Pseudomonas* species are psychrotrophic, able to thrive at temperatures below 7 °C, with a temperature range from 0 °C to 40 °C. Numerous species of *Pseudomonas* bacteria exhibit diverse roles, characterized by fast multiplication, adaptability to low-temperature growth, protein degradation to yield amino acids, and a pronounced capacity for decay. *Pseudomonas* spp. can infect fresh aquatic items, making them sticky on the surface, producing a disagreeable smell, and finally causing food to rot. *P. fluorescens*, *P. aeruginosa*, *P. syringae*, *P. fragilis*, *P. lunata*, *P. azogenes*, *P. marginata*, and *P. malodorans* are prevalent in refrigerated aquatic products [20,21]. For example, the specific spoilage microorganisms in tuna and turbot are dominated by *P. fluorescens* [22]. *P. malodora* is one of the important spoilage bacteria of freshwater fish. Zhuang et al. [23] isolated *P. malodorata* from grass carp fillets and found that the bacterium had a strong ability to absorb and metabolize free amino acids, which promoted the spoilage of grass carp. In addition, it was shown that 64.85% of the *Pseudomonas* isolated from hybrid grouper were *P. azogenes*, which had a greater adverse effect on the odor and flavor of refrigerated grouper [24]. Another study found that *Pseudomonas* is not only a dominant spoilage organism in cultured fish, but it also grows dominantly in refrigerated shrimp products [25]. A partial compilation of *Pseudomonas* species in various aquatic products is presented in Table 2.

### 2.2. Cold Adaptation Mechanism of Pseudomonas spp.

**Figure 1:**
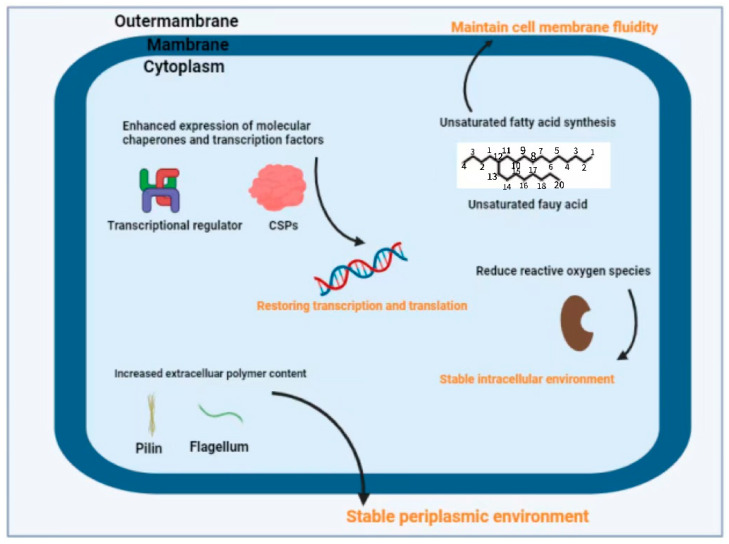
Cold adaptation mechanisms of *Pseudomonas* spp.: ① enhanced expression of molecular chaperones and transcription factors, restoring transcription and translation; ② by synthesizing unsaturated fatty acids to maintain cell membrane fluidity; ③ reducing reactive oxygen species to maintain a stable intracellular environment; ④ increased extracellular polymer content to maintain a stable periplasmic environment.

Substantial changes in *Pseudomonas* cell physiology occur under low-temperature conditions, primarily through the disruption of cell membranes, interference with protein folding, and inhibition of transcription and translation processes [33]. Rapid chilling processes induce phospholipid phase separation within the lipid bilayer, thereby increasing membrane permeability and ultimately leading to cell death [34]. Some *Pseudomonas* spp. are cold-adapted microorganisms that grow in low temperature environments for a long time, with a series of special structures and complex regulatory mechanisms [35], for example, regulating the fatty acid composition of the cell membrane to maintain fluidity at low temperature, production of enzymes that can maintain catalytic activity at low temperature, over-expression of cold shock proteins (CSPs) to ensure the normal metabolism of the cell at low temperature, and forming alginate sugar with low-temperature protection [36,37,38]. The cold adaptation mechanism of *Pseudomonas* is shown in Figure 1.

#### 2.2.1. Regulation of Cell Membranes

The consistent fluidity of cell membranes is crucial for sustaining the normal physiological functions of microorganisms [39]. In low-temperature environments, microorganisms adapt by preserving the fluidity of their cell membranes, which is essential for their survival. This adaptation involves adjusting the fatty acid composition of the membranes, in which branched-chain and polyunsaturated fatty acids are crucial. The regulation of fatty acid desaturase activity is instrumental in this process, promoting the desaturation of fatty acids and thereby preserving membrane fluidity [40,41]. Furthermore, various regulatory systems, including both double-component and single-component mechanisms, exert influence over membrane fluidity by modulating the activity of key proteins (such as desaturases) [42]. Research has shown that increasing the content of unsaturated fatty acids in membrane lipids is beneficial, and that increasing the proportion of branched-chain fatty acids in membrane lipids is also helpful. This effect occurs within a certain range of carbon chain length and number of double bonds. These changes are beneficial for maintaining the fluidity and permeability of cell membranes, especially so in low-temperature environments [43,44].

(i) Branched-chain fatty acids

The normal cell membrane is liquid and disordered due to the existence of unsaturated fatty acids or terminal branched fatty acids, counteracting the tight packing and ordered arrangement of acyl chains imparted by linear saturated acyl chains in the lipid bilayer [45]. Previous research indicates that methyl groups exert a notable disruptive influence on acyl chain accumulation, thereby impacting cell membrane fluidity. Specifically, the methyl branch chain of allofatty acid resides farther from the fatty acid terminal, thereby facilitating heightened membrane fluidity. This phenomenon correlates with a lower phase transition temperature necessary for maintaining normal membrane function [46]. Furthermore, research indicates that the cell membrane composition of Antarctic cold-adapted *Flavobacterium* spp. predominantly comprises branched-chain fatty acids and unsaturated fatty acids. This composition serves to uphold the requisite fluidity of the cell membrane under low-temperature conditions [44]. In the cell membrane of *Cryptobacter glacialis*, approximately 70% of the fatty acids are comprised of straight-chain monounsaturated fatty acids and branched-chain fatty acids. Temperature fluctuations significantly impact the balance between these two types of fatty acids within microbial cell membranes. *Pseudomonas* spp. that were found in the Antarctic polar region by Morozova et al. [47] also adapted to the cold environment by regulating the branched-chain fatty acid composition of the cell membrane. Specifically, as temperature increased, there was a notable decrease in the proportion of branched-chain fatty acids relative to straight-chain fatty acids [48]. On the whole, the synthesis of branched-chain fatty acids emerges as a pivotal factor in microbial cold adaptation.

(ii) Polyunsaturated fatty acids

Polyunsaturated fatty acids were detected at low temperatures when studying temperature-driven adaptation in glacier-dwelling bacteria [48]. In cold environments, the polar algae *Chlamydomonas reinhardtii* RCC2488 synthesizes significant quantities of polyunsaturated fatty acids to uphold cell membrane fluidity [40]. Bao et al. [49] reported that *P. fragilis* D12 could maintain cell membrane fluidity by shortening the average chain length of fatty acids when the temperature was reduced from 30 °C to 15 °C. The stability of the extracellular environment was maintained by increasing the expression level of hair proteins, improving the adhesion capacity and increasing extracellular polymer content. As the temperature further decreased from 15 °C to 4 °C, most of the genes related to fatty acid degradation were downregulated, while the key genes related to unsaturated fatty acids synthesis were upregulated. Under this temperature change, *P. fragilis* D12 maintained cell membrane fluidity by boosting the unsaturated fatty acids content. Li et al. [50] also found that the unsaturated fatty acid content of *P. putida* B6-2b was 56% at 30 °C and increased to 89% at 5 °C. The unsaturated fatty acids contents in the membrane increased with the decrease in temperature.

(iii) Fatty acid desaturases

Fatty acid desaturases are the main enzyme for fatty acid desaturation, and their synthesis is influenced by temperature stress. Notably, Ole1 is an iron-dependent fatty acid desaturase in *Saccharomyces cerevisiae*, playing a crucial role in regulating unsaturated fatty acid biosynthesis in response to temperature fluctuations [51]. Tae-Rim Choi et al. found that in novel *Pseudomonas* psychrophila, overexpression of the Δ-9 fatty acid desaturase gene resulted in increased levels of unsaturated fatty acids, leading to increased membrane fluidity [52]. Hybrid histidine kinases induced elevated expression levels of Δ12/Δ15 fatty acid desaturase and glycerol 3-phosphate dehydrogenase at lower temperatures, resulting in increased biosynthesis of polyunsaturated fatty acids by the respective enzymes [53], proving that a certain level of activity of the desaturase enzyme is necessary for the membrane to adapt to low temperatures. In addition, no matter how the ambient temperature changes, the addition of more unsaturated fatty acids with similar properties is vital for maintaining appropriate membrane lipid fluidity [54].

Broadly speaking, the modulation of microbial cell membrane fluidity constitutes a intricate regulatory mechanism responsive to temperature variations. The production of unsaturated fatty acids by fatty acid desaturase enzymes is crucial for the survival of *Pseudomonas* spp. under cold stress. Some cold-tolerant strains such as *Pseudomonas* sp. AMS8 and *Pseudomonas* sp. A3 have been shown to produce significant amounts of monounsaturated fatty acids when exposed to low temperatures [55]. To investigate the cold stress response, functional genomics were employed using the bacterial model organism *P. putida* KT2440 [56]. Transcriptome sequencing and proteome peptide profiling of KT2440 revealed upregulation of the valine degradation pathway into branched-chain fatty acids during growth at low temperatures.

#### 2.2.2. Low Temperature Protease

Cryogenic microorganisms can synthesize proteases with catalytic activity under low temperature to maintain their normal physiological activities [57]. The mechanisms by which molecules adapt to low temperatures vary from enzyme to enzyme, but most have the same mechanisms. These changes involve a reduction in the number of hydrogen bonds, changes in salt bridges, proline, and arginine content, and the interaction of aromatic molecules [58]. Lowering the temperature had the opposite effect on enzyme kinetics, with enzyme activity reduced half with a temperature drop of 10 °C. Cryoproteases maximize cryogenic activity by destroying structures that contain the active site and the entire molecule, due to the weakening of intramolecular bonds (electrostatic interactions, subunits, disulfide bonds) as well as the elimination of stability factors, leading to improved dynamics of the active site [59]. Enhanced enzyme flexibility can be attributed to various factors, including increased surface area, core hydrophobicity, and precise alignment of amino acids. Studies suggest that the existence and arrangement of certain amino acids in the enzyme structure play a pivotal factor in the stability and activity of enzymes. Öten et al. [60] observed that proline (Pro) and glycine (Gly) residues altered conformational changes in the three-dimensional (3-D) structure of proteins, thereby promoting the adaptation of enzymes to cold conditions. The high catalytic efficiency of cold-adapted enzymes at low temperature reflects the adaptability of cold-adapted microorganisms to low-temperature environments. Enzymes with low-temperature catalytic activity can improve their stability and catalytic activity through structural modification. Proteomic studies have shown that transport proteins located in the membrane can counteract low diffusion rates by enhancing the uptake of essential solutes, nutrients, and peptides necessary for peptidoglycan synthesis [61]. Similar findings were observed in *P. aeruginosa* [62], where specific channel proteins were expressed to facilitate nutrient absorption. The study by Yang et al. [63] discussed the cold-adapted proteases in *Pseudomonas,* which are important for maintaining the physiological activities of *Pseudomonas* at low temperatures.

#### 2.2.3. Cold-Adapted Proteins

Cold-adapted proteins are a set of proteins that are upregulated by microorganisms in cold environments to help them survive in low temperatures. These proteins play a pivotal role in sustaining the regular physiological activities of organisms under low-temperature conditions [64]. Microorganisms produce a transient cold-induced response to a sudden drop in ambient temperature, expressing a variety of cold-stressed proteins [6]. CSPs and the ice-binding proteins (IBPs) are two strategies for *P. aeruginosa* to adapt to frigid environments [65,66].

(i) CSPs

The CSP family is widely distributed among organisms, characterized by low molecular weight and high levels of conservation. It encompasses various protein domains, notably the S1 domain, rich in residues associated with RNA binding and protein synthesis; the S12 domain, responsible for metal ion binding; the S17 domain, primarily involved in rRNA binding and structural components of ribosomal activity; and the S28e domain, which serves as a structural component [67,68]. The gene encoding CSPs was expressed immediately after cold shock [69]. CSPs are a transcription anti-terminator or translation enhancer that destroy the stability of RNA secondary structure at low temperature. This instability of RNA secondary structure can prevent premature termination of transcription during cold shock. A study by Khan et al. [70] looked at the levels of expression of CSPs from *P. fluorescens* MTCC 35 (MW 8 kD) and cold-resistant proteins (MW 4kD) from *P. fluorescens* mutant CRPF37 between 103 °C and 14 °C. The expression of CSPs and cold-resistant proteins increased with the decreasing temperature, and the induced protein synthesis rate reached its maximum at 10 °C.

(ii) IBPs

**Figure 2:**
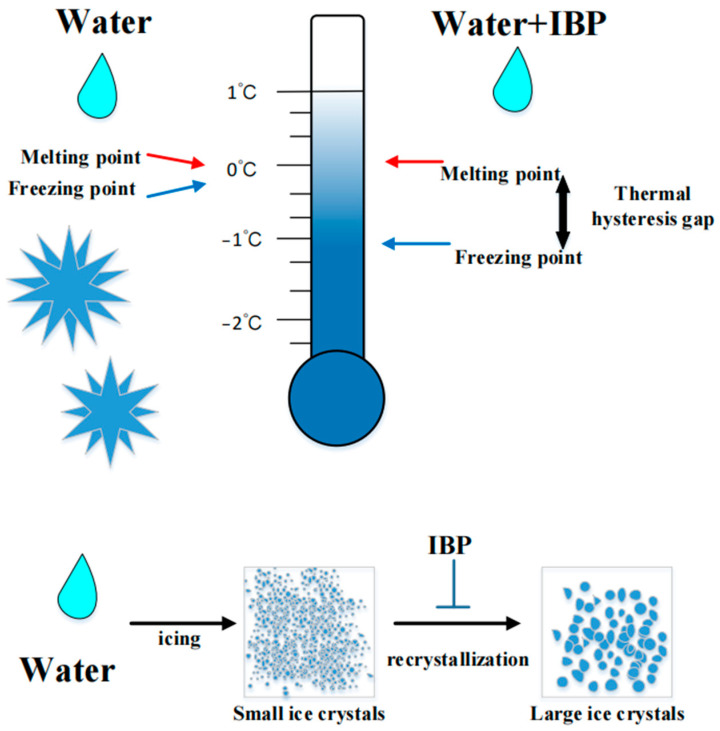
Freezing point lag of mechanism: ice-binding proteins make ice crystals larger, IBPs: ice-binding proteins.

IBPs include antifreeze proteins (AFPs) and ice nucleation proteins (INPs). Although IBPs share similar functions, their sequence, structure, and activity characteristics vary significantly. After encountering IBPs, the freezing point of water changes. In the absence of IBPs, the freezing and melting points of an aqueous solution coincide, resulting in numerous small ice crystals. Introduction of IBPs into the solution results in a slight lowering of the freezing point and a slight raising of the melting point of water, accompanied by an increase in ice crystal volume. The phenomenon of thermal hysteresis (TH), reflected in the temperature difference between the freezing point and the melting point, is chiefly responsible for lowering the water’s freezing point below its melting point [71]. A graphical summary of the mechanism is shown in Figure 2.

IBPs such as AFPs inhibit ice diffusion by lowering the freezing point and inhibiting ice recrystallization [72]. Previous research has indicated differential gene expression in response to varying cold temperatures. Firdaus-Raih et al. [73] analyzed nine AFPs and noted their survival under various extreme cold conditions and exposure durations. Antifreeze proteins exhibiting frost resistance have been found in several species of *P. psychrotrophs*, including *P. sychrotropha*, *P. fluorescens*, and *P. putida* [74,75]. The antifreeze gene afpA from *P. putida* GR12-2 was successfully cloned into *E.coli*. Compared with the natural protein, the protein showed low levels of antifreeze and ice nucleation activity. Among various cold-adapted microorganisms, including *P. syringae*, *P. borealis*, *Xanthomonas* and *P. fluorescens*, there are INPs that are classified as IBPs and have a conserved domain (16-residue ice nucleation motifs of 50–80 tandem repeats) [76]. These domains are positioned predominantly on the bacterial cell surface or exterior. The primary biological role of INPs lies in providing freezing protection through the generation of extracellular ice crystals, thereby enhancing the survival prospects of bacteria. Unlike AFPs, INPs are very large, with a polymer range of 120 to 150 kDa. AFPs play a crucial role in mitigating freezing damage by impeding the growth of larger ice crystals, whereas INPs facilitate the initiation of ice crystal formation [77]. In a study of *P. fluorescens* derived from insects, a comparative analysis of hyperactive AFPs and INPs was performed; it was observed that both anchored inclusion complexes and ice-like patterns exhibited comparable efficacy in binding to proteins on ice surfaces and promoting ice nucleation [78].

### 2.3. Regulatory Mechanisms of Biofilm Formation

**Figure 3:**
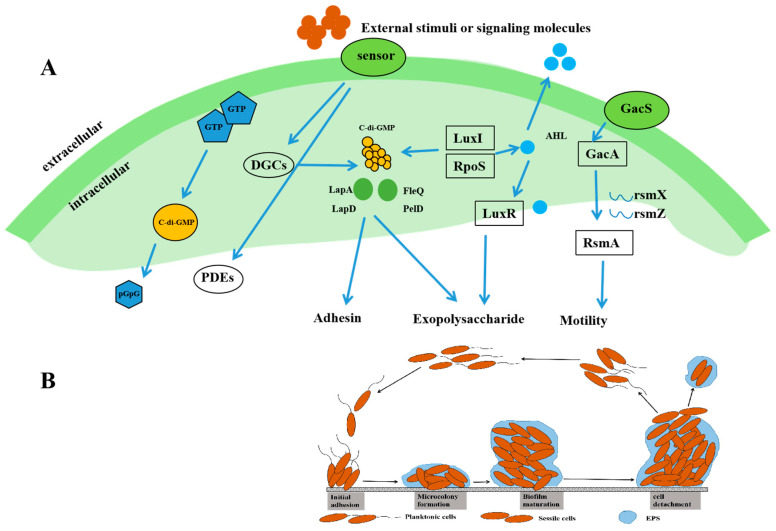
(**A**) The regulatory mechanism of biofilm formation in *Pseudomonas* spp. is composed of three pathways, namely, the C-di-GMP regulatory pathway, the quorum sensing system, and the TCS. (**B**) Four stages of the biofilm formation process. GTP: DGCs: diguanylate cyclase, C-di-GMP: cyclic di-guanosine monophosphate, PDE: phosphodiesterases, LapD: receptor protein, LapA: adhesion protein, AHLs: N-acyl-homoserine lactones, EPs: extracellular polymers.

Biofilms of *Pseudomonas* spp. consist mainly of bacteriophage and extracellular polymers (EPs) [79]. EPs account for 50% to 80% of the organic matter content in biofilm, which is mainly composed of polysaccharides, proteins, lipids, extracellular DNA, and extracellular RNA. There are four stages in the sequential process of biofilm formation. The first step is the migration of cells and their adhesion to surfaces; the second step is micro-colony formation and exudation of EPs from cells; the third step is the maturation stage; the fourth step is cell detachment [80]. The process of biofilm formation [81] by spoilage bacteria in aquatic products involves the expression of various genes, the adhesion of proteins, and the regulation of regulatory factors and signaling molecules. The following is a brief description of the regulatory mechanism of biofilm formation by *P. aeruginosa* in aquatic products. The mechanism diagram is shown in Figure 3.

#### 2.3.1. Cyclic Di-Guanosine Monophosphate (C-di-GMP) Signaling Factor

The second messenger C-di-GMP is ubiquitously found in bacteria and regulates the formation of biofilms as well as cell division, which is important for the formation of biofilms in Gram-negative bacteria [82]. The general rule is that when the intracellular C-di-GMP content is high, the organism exists in biofilm form; when the content is low, it exists in planktonic state. C-di-GMP is synthesized from two molecules of GTP catalyzed by diguanylate cyclase (DGC) and decomposed by phosphodiesterases (PDE), which have conserved catalytic sites Gly-Gly-Asp-Glu-Phe (GGDEF) and Glu-Ala-Leu (EAL), respectively [83]. In *P. fluorescens*, intracellular C-di-GMP is decomposed when the reaction regulator promotes PDE synthesis, and low concentrations of C-di-GMP inhibit the synthesis of adhesion proteins. *P. fluorescens* has C-di-GMP receptor protein LapD, protease LapG, and adhesion protein LapA. When the concentration of C-di-GMP is low, LapG can degrade LapA and make the organism lose adhesion, precluding the growth of biofilm. High concentrations of C-di-GMP form a complex with the receptor protein LapD, which is able to bind to LapG to inactivate it, thus promoting synthesis of the adhesion protein LapA [84].

#### 2.3.2. Quorum Sensing (QS) Systems

QS is a communication system among bacterial cells that regulates behaviors of bacterial groups, such as bioluminescence, biofilm formation, and toxin production [85]. According to the different signal molecules, QS can be divided into different regulatory pathways: the LuxI/R signal system, which is the QS system of most Gram-negative bacteria, with N-acyl-homoserine lactones (AHLs) as signal molecules, and the small-molecule peptide-type signal system, which is mediated by oligopeptides (autoinducing peptides, AIPs) as signal molecules and exists in Gram-positive bacteria. The LuxS/AI-2 signal system is a QS system mediated by furanboronic acid diesters (Autoinducer-2, AI-2) [86]. In addition, other signaling molecules such as diketopiperazines (DKPs) can simultaneously regulate QS between and within species [87]. In *P. fluorescens*, the QS regulatory system features LuxI/R-type and LuxS/AI-2-type signaling. Liu et al. [88] found that RpoS regulatory factors could directly regulate the synthesis of AHL factors, thereby enhancing the spoilage ability of bacteria. The biofilm characteristics of co-cultured *P. fluorescens* and *S. baltica* wer studied; the amount of biofilm of *P. fluorescens* and *S. baltica* was lower than that of single *P. fluorescens*, indicating a certain competition between the two co-cultures. This may be because *S. baltica* does not produce AHLs but consumes or inhibits AHLs produced by *P. fluorescens*, thereby inhibiting the production of *S. baltica* biofilms [89].

#### 2.3.3. Two-Component Control System (TCS)

The TCS is a regulatory mode in which bacterial cells convert environmental signals into chemical signals, consisting of a histidine kinase (HK) that senses environmental signals and a regulatory protein (RP) coupled to it. In *P. putrefaciens fluorescens*, the two-component regulatory system is GacS-GacA. Cheng et al. [90] used transcriptional profiling of GacS mutants of *P. fluorescens* to show that this TCS promoted the synthesis of extracellular polysaccharides and the expression of genes related to C-di-GMP signaling molecules. Similarly, in *P. fluorescens* F113, disruption of the GacSGacA system led to increased motility and disruption of biofilm synthesis in the bacterium [91]. The transcription of RNA small molecules RsmA and RsmZ is activated by the GacS-GacA system, which inhibits the transcription of motor genes, promotes the synthesis of extracellular polysaccharides in biofilms, and ultimately promotes the synthesis of biofilms.

## 3. The Spoilage Indicators of *Pseudomonas* spp.

Sensory scores and total viable count (TVC) are commonly used and reliable indicators for assessing the freshness of aquatic products. However, relying solely on sensory scores and TVC is insufficient to directly assess the impact of *Pseudomonas* spp.’s influence on aquatic products. The effect of protein degradation and lipid oxidation on the freshness of aquatic products is significantly increased in the presence of *Pseudomonas* spp. During spoilage, *Pseudomonas* spp. produce various metabolites, including volatile compounds, which reflect both the spoilage potential of these bacteria and the overall freshness of the product. These metabolites can serve as indicators for a more thorough assessment of the quality of aquatic products. Indicators primarily include volatile compounds (biogenic amines, trimethylamines, alcohols, and total volatile base nitrogen), thiobarbituric acid, and *K*-value.

### 3.1. Volatile Organic Compounds (VOCs)

*Pseudomonads* spp. can utilize organic compounds in aquatic products to produce large quantities of volatile organic compounds (VOCs). These VOCs are responsible for the deterioration of the odor and flavor of aquatic products and have a significant impact on the organoleptic and nutritional value of aquatic products [31]. VOCs include biogenic amines (BAs), trimethylamines (TMA), alcohols, ketones, aldehydes, esters, amines, alkanes, and organic acids (Table 3). Currently, there are many techniques for the analysis of VOCs in aquatic products, such as chromatographic techniques [92], omics analysis, headspace solid-phase microextraction (HS-SPME) combined with gas chromatography–mass spectrometry (GC-MS) [93], etc. Some VOCs are listed in Table 3.

#### 3.1.1. BAs

Most of the time, BAs are produced when certain free amino acids are decarboxylated or when ketones and aldehydes are aminated or transaminated [94]. Numerous investigations have identified the existence of eight major types of BAs in aquatic products, namely phenylethylamine, cadaverine, putrescine, spermine, tyramine, tryptamine, spermidine, and histamine [94,95,96]. Increased BAs in aquatic products not only have a negative impact on flavor, but large accumulations in the human body can lead to poisoning and serious health problems such as headaches, edema, diarrhea, etc. and can even be life-threatening [97]. When proteins in aquatic products are catabolized into small molecules of peptides and amino acids by proteases secreted by *P. aeruginosa*, ornithine and arginine synthesize putrescine under the action of ornithine decarboxylase (ODC) and arginine decarboxylase (ADC) [98]. Many bacterial species, including *P. putida*, *P. fluorescens*, and *Aeromonas* spp., are capable of producing histamine [99]. Histamine is produced by the action of histidine decarboxylase [100], and lysine produces cadaverine by the action of lysine decarboxylase. BAs can be used as a marker of food quality, and a variety of analytical methods have been developed in recent years for the detection of BAs [32], including high-performance liquid chromatography (HPLC) [101], capillary gas chromatography (GC) [102], capillary zone electrophoresis (CZE), and ion-exchange chromatography (IEC) [96]. Xie et al. [103] inoculated *P. fluorescens* into salmon fillets, and the results of preservation experiments at 4 °C and 30 °C showed that amines and putrescine were considered to be the main BAs in salmon samples. *P. fluorescens* is an active producer of the above BAs. With the increase in storage time, the yield of cadaverine and putrescine increased gradually. A more significant difference was observed between the inoculated and uninoculated samples at 4 °C than at 30 °C, indicating that *P. fluorescens* maintained high decarboxylase activity at low temperatures.

#### 3.1.2. TMA

Protein degradation in aquatic products releases amino acids. *Pseudomonas* spp. decarboxylates these amino acids to form TMA [104]. TMA is a harmful VOC with negative effects on human health [105]. Once the human body inhales a certain amount of TMA, many harmful reactions such as dyspnea, headaches, pulmonary edema, nausea, and upper respiratory tract irritation can occur [106]. A number of studies have shown that dimethylamine (DMA), formaldehyde (FA), and TMA in aquatic products originate from the decomposition of TMAO [107]. TMA has generally been studied extensively, as it is used as an indicator of fish spoilage due to its fishy odor. In recent years, HPLC, GC-MS and ion mobility spectrometry (IMS) have been widely used for the detection of TMA. Wang et al. [16] inoculated *P. fluorescens* on tuna fillets. After 6 days of storage at low temperature, the abundance of *Pseudomonas* spp. increased gradually, and trimethylamine oxide was rapidly decomposed into trimethylamine.

#### 3.1.3. Total Volatile Base Nitrogen (TVB-N)

TVB-N relates to the breakdown of proteins in aquatic products by microbes and enzymes, producing low-base volatile nitrogen compounds such as amines and ammonia. TVB-N content is closely related to the extent of corruption of aquatic products and is one of the most commonly used indicators to assess their freshness. It has been shown that TVB-N levels are positively correlated with the total bacterial count, effectively reflecting the abundance of spoilage bacteria and the overall quality of the product [108]. The yield factor of spoilage metabolites (Y_TVB-N/CFU_) is an important way to measure how much bacteria are spoiling a sample [99]. It shows how many spoilage metabolites one unit of bacteria makes at the end of the spoilage process. The Y_TVB-N/CFU_ value can be used as a quantitative standard to express the degree of spoilage by spoilage bacteria, which can better reflect the degree of spoilage of aquatic products. Higher Y_TVB-N/CFU_ values indicate a greater capacity for spoilage, reflecting the efficiency of specific spoilage organisms (SSOs) in degrading fish proteins into nitrogenous compounds that contribute to off-flavors and odors. In studies involving *Pseudomonas* and *S. thermophilus* isolated from refrigerated raw tuna, it was found that *Pseudomonas* species played a significant role in the spoilage process; the results showed that *Pseudomonas* played the most important role. The Y_TVB-N/CFU_ value of *P. fluorescens* in salmon was observed to be higher in samples stored at low temperatures than in samples stored at high temperatures [103].

**Table 3:** Volatile compounds (partially displayed) detected in aquatic products.

Categories	Representatives	Source	Hazard	References
Alcohols	1-penten-3-ol, ethanol, methyl mercaptan, 0-methyl-4-butanol, isopropanol, 37-ethyl-0-hexanol, 4-penten-8-ol, etc.	Oxidation of polyunsaturated fatty acids	Fishy, fatty, mushroomy, or grassy flavor	[93,109]
Aldehydes	Nonanal, hexanal, decanal, 3-methylbutyraldehyde, trans-2-octenal, 8-methylbutyraldehyde and lauric aldehyde, etc.	Alkoxy radicals and derivatives of unsaturated fatty acids	Causes nausea, vomiting, abdominal pain and other digestive symptoms	[110]
Ketones	2-octanone, 4-methyl-2-pentanone, 2-pentanone, 2-heptanone, n-nonanone, 2-undecanone, etc.	Oxidation or degradation of the unsaturated fatty acids and degradation of amino acids	Toxic, with a pungent odor Affects appetite, causes headaches	[111]
Esters	Butyl butyrate, isobutyl isobutyrate, ethyl 2-methylbutyrate, etc.	Composed of the reaction products of alcohols and acids resulting from acid–alcohol condensation	Has a strong smell of corruption, may cause allergic reactions or gastrointestinal discomfort	[112]

### 3.2. Thiobarbituric Acid (TBA)

Aquatic products are high in unsaturated fatty acids, which can be easily oxidized. Over time, these fatty acids degrade into low molecular weight compounds like ketones, aldehydes, and carboxylic acids. These oxidation products can alter the odor, color, texture, and nutritional value of aquatic products. Lipid oxidation is closely linked to the activity of lipase enzymes produced by spoilage bacteria. The TBA value is a useful indicator for assessing the extent of lipid oxidation and the freshness of aquatic products [112]. In a study of the cold chain logistics of Spanish mackerel, the total DNA of microorganisms was extracted and high-throughput testing was carried out. It was found that *Pseudomonas* spp. were the dominant spoilage bacteria of Spanish mackerel, and TBA values showed an upward trend with transportation time [113].

### 3.3. K-Value

**Figure 4:**
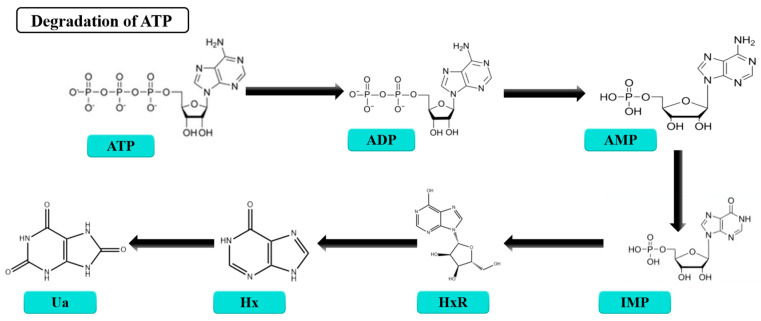
The ATP degradation process. ATP: adenosine triphosphate, ADP: adenosine diphosphate, AMP: adenosine monophosphate, IMP: inosine 5′-monophosphate, HxR: inosine, Hx: hypoxanthine, Ua: uric acid.

The decomposition of adenosine triphosphate (ATP) plays a central role in the postmortem changes of aquatic products. The ATP breakdown process, which progresses from ATP to adenosine diphosphate (ADP), adenosine monophosphate (AMP), inosine monophosphate (IMP), and inosine (HxR), reflects the initial biochemical changes in aquatic products prior to bacterial growth [114] (Figure 4). However, the conversion of HxR to hypoxanthine (Hx) is accelerated by the activity of spoilage bacteria such as *Shewanella putrefacien*. The *K*-value is defined as the percentage ratio of the total amounts of HxR and Hx to the total ATP-related compounds. The formula used to calculate the *K*-value is as follows:

$$K-\mathrm{value}\;(\%)=\frac{\mathrm{HxR}\;+\;\mathrm{Hx}}{\mathrm{ATP}\;+\;\mathrm{ADP}\;+\;\mathrm{AMP}\;+\;\mathrm{IMP}\;+\;\mathrm{HxR}\;+\;\mathrm{Hx}}\times \;100$$

A higher *K*-value signifies more rapid ATP breakdown. This value serves as an important indicator of aquatic products’ freshness. For example, the *K*-value of filleted ray fish showed an exponential increase during storage, signaling the progression of both freshness loss and spoilage. Similarly, the *K*-value of tilapia fillets was strongly linked to both sensory acceptability and storage time [115]. Because of its psychrophilicity, *Pseudomonas* spp. was the dominant spoilage bacteria affecting the preservation of puffer fish, and the *K*-value of puffer fish showed an upward trend during storage [116].

## 4. Technologies for Detection of *Pseudomonas* spp.

Traditional methods for the detection of *Pseudomonas* spp. include microscopic observation, plate isolation and culture, and biochemical reactions. However, these approaches are often characterized by lengthy processing times and limited sensitivity and accuracy. With the current rapid development of molecular biotechnology, molecular detection techniques have been widely used for the detection of *Pseudomonas* spp. Cao et al. studied the detection of *Pseudomonas* in aquatic products using visual color chips based on asymmetric multiple polymerase chain reactions and nucleic acid hybridization [117]. The detection techniques for *Pseudomonas* spp. in aquatic products mainly include recombinase polymerase amplification (RPA), polymerase chain reaction (PCR), loop-mediated isothermal amplification (LAMP), next-generation sequencing (NGS), and fluorescence in situ hybridization (FISH).

### 4.1. RPA

RPA is a fast, highly sensitive, and specific nucleic acid amplification method that has been effectively applied for the detection of *Pseudomonas* spp. This method utilizes recombinase enzymes along with accessory proteins to unwind and anneal primers to the target DNA or RNA, enabling efficient amplification [118]. Tran et al. [119] used multiplex RPA technology to reliably detect foodborne bacteria including *P. aeruginosa* and *S. aureus* in milk, juice, and bottled water. Soliman et al. [120] used RPA technology to detect pathogenic viruses in carp, and Tang et al. [121] used RPA technology to detect *Vibrio cholerae* in seafood. It is a fast, simple and sensitive detection method. By combining CRISPR/Cas12a and RPA technologies, specific crRNAs and RPA primers were designed to target the ATPase gene for quick and sensitive detection of iris viruses in large yellow croaker [122]. Overall, RPA offers significant potential for detecting spoilage bacteria in aquatic products. However, its limitations (Table 4) must be taken into account when selecting the most suitable amplification method for a particular application.

### 4.2. PCR

**Figure 5:**
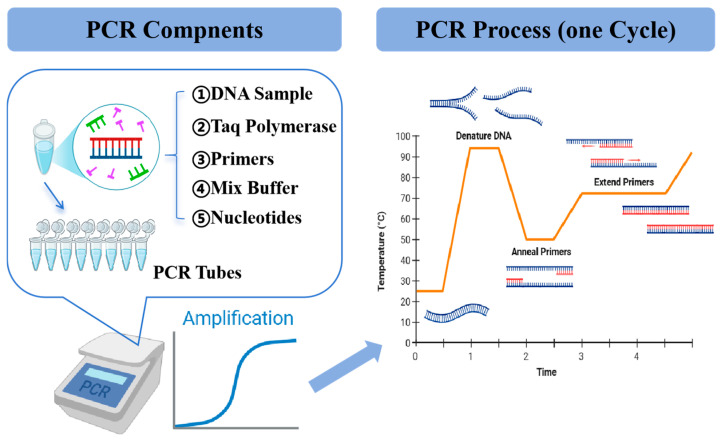
PCR experimental technique and principle analysis diagram: (1) denaturation, heating the double-stranded DNA template to dissociate it; (2) annealing, where short DNA molecules called primers bind to flanking regions of the target DNA; (3) extension: DNA polymerase extends the 3’end of the primer along the template chain. Blue represents the DNA template, red represents the primer, and arrows indicate the amplification direction.

PCR is a commonly used laboratory technique, which is widely used in the detection of spoliage bacteria in aquatic products, as well as many other fields of research and medicine. Nair et al. used a molecular method with multiplex PCR to rapidly detect Bacillus and Pseudomonas in aquatic products [123]. PCR amplifies specific genetic sequences using a polymerase enzyme and targeted primers (Figure 5). For example, it can identify *P. syringae* by employing primers that correspond to various gene regions, such as the 16S-23S rDNA intergenic spacer region [124]. Li et al. [125] established a multiplex PCR detection technology that enables the simultaneous detection of *Vibrio parahaemolyticus*, *Listeria monocytogenes*, *Streptococcus flexneri*, *P. putida*, *E. coli*, *V. vulnificus*, and *V. alginolyticus* contamination in seafood. This method is highly effective for the analysis and detection of pathogenic microorganisms in various seafood products. In addition to seafood, it can also be used in other food samples [126]. It is a rapid, simple, and sensitive detection method. Overall, PCR is a rapid, straightforward, and sensitive method for detection. While it is a powerful and widely employed tool for identifying spoilage bacteria in aquatic products, it is essential to weigh both its strengths and limitations (Table 4) when planning and interpreting experiments.

### 4.3. LAMP

LAMP is a fast, highly sensitive, and specific DNA amplification technique that has become widely utilized for detecting spoilage bacteria in aquatic products. The principle of this method is to use two pairs of different primers to amplify the target sequence of DNA by strand displacement polymerase (exempelvis Bst DNA polymerase) under isothermal conditions (usually 65 °C) [127]. Through this mechanism, extremely low concentrations of nucleic acid molecules can be detected in just tens of minutes without the use of expensive instruments. For example, LAMP can use primers based on different genes to detect clove false antibodies, such as type III effector genes or enolase genes [128]. Ferrusca Bernal et al. [129] used isothermal amplification combined with reverse line blot hybridization to detect *P. aeruginosa*. This approach enabled the identification of *P. aeruginosa* genetic material by amplifying and hybridizing conserved sequences within the 16S ribosomal gene. Zhou et al. [130] have developed a real-time fluorescent ring-mediated isothermal amplification assay (on-chip LAMP) integrated on a microfluidic disk chip, which can be used to rapidly and simultaneously detect 10 pathogenic bacteria (including *Pseudomonas* spp.) in aquatic animals.

### 4.4. NGS

**Figure 6:**
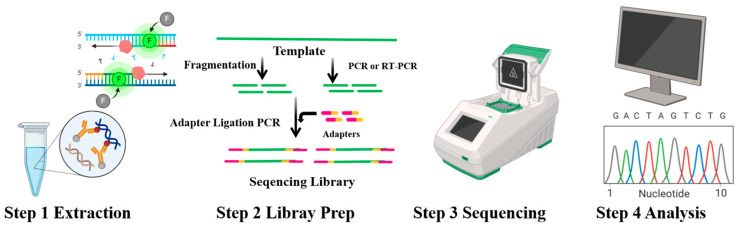
NGS experimental technique and principle analysis diagram. Blue represents the DNA template, red represents the primer, and arrows indicate the amplification direction.

NGS is a high-throughput technology for DNA/RNA sequencing, enabling rapid and accurate analysis of large amounts of genetic material in a single experiment (Figure 6). This technology has transformed genomics by allowing researchers to examine entire genomes, exomes (protein-coding regions), and transcriptomes (the complete set of RNA molecules in a cell or group of cells) with unparalleled precision. NGS is widely applied in diverse areas, including genomic sequencing, gene expression profiling, metagenomic analysis, genetic variation studies, and gene function research. It has become an essential tool in genetics, genomics, and medicine, driving numerous scientific advancements and breakthroughs [131]. In the context of aquatic products, NGS-based metagenomic analysis enables the examination of microbiome composition and function, providing insights into the collective genetic information of microorganisms residing on or within these products. This approach holds great potential for detecting *P. syringae* and other co-existing pathogens. For example, NGS can identify *P. syringae* by sequencing its entire genome or specific regions, such as the 16S rRNA gene or multilocus sequence typing (MLST) loci [132]. Overall, NGS is a powerful tool for detecting spoilage bacteria such as *P. syringae* in aquatic products. However, it is important to carefully consider both its strengths and limitations (Table 4) when designing and conducting related studies.

### 4.5. FISH

**Figure 7:**
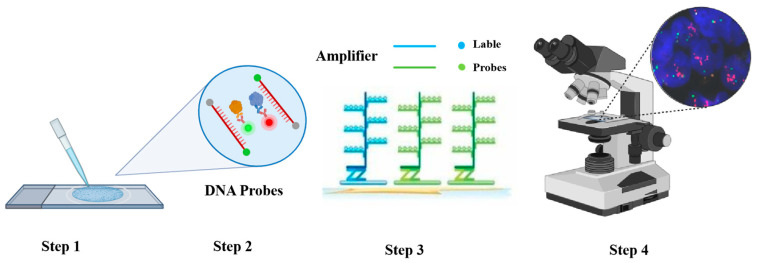
FISH experimental technique and principle analysis diagram. In step 1, the red single strand represents the mRNA, and the red and green fluorescent dots represent the target probe, The red dots in Step 4 represent the target DNA.

FISH is a molecular biology method that utilizes fluorescently labeled probes to identify and localize specific DNA or RNA sequences within cells or tissues (Figure 7). This technique works by hybridizing complementary probes, each tagged with distinct fluorophores, to the target sequences. The distribution and position of these sequences can then be visualized using a fluorescence microscope. Wang et al. [133] used the ssDNA aptamer as a probe for the rapid FISH method to detect *P. aeruginosa*, which proved the value of aptamer FISH for bacterial detection. It can provide reliable, rapid, and cheap detection of *P. aeruginosa* in a laboratory equipped with common equipment. Alessandro et al. [134] used FISH to detect probiotics in the gut of tilapia fish; their results suggest that FISH technology is a potential tool for detecting pathogenic bacteria in aquatic products. Overall, FISH is a powerful technique for the detection of spoilage bacteria in aquatic products; however, it has certain limitations (Table 4) that must be taken into account when choosing the most suitable method for a particular study.

**Table 4:** Comparison of detection methods for *Pseudomonas* spp.

Methods	Principle	Advantages	Disadvantages	Reference
RPA	The rapid amplification of target DNA is achieved by the synergistic effect of DNA recombinase and polymerase	High sensitivity, high specificity, rapid turnaround time, easy to use	Limited multiplexing, low throughput, poor stability, high cost.	[135]
PCR	Amplifies specific genetic sequences using a polymerase enzyme and targeted primers	Quantitation possible, sensitivity, specificity, speed, versatility	PCR system affects the effectiveness, complexity, false positives, high cost	[136]
LAMP	Four primers are designed for the six regions of the target gene, and the amplification reaction is carried out using the strand displacement DNA polymerase under constant temperature conditions	High sensitivity, specificity, rapid turnaround time, simplicity	Limited multiplexing, poor performance with complex DNA templates, inability to detect DNA deletions or insertions, limited commercial availability	[127]
NGS	Improves sequencing speed and reduce costs by sequencing a large number of DNA fragments in parallel	High throughput, high accuracy, multiplexing, large scale, high resolution, versatility	Technical expertise, sample quality, data analysis, limited access	[132]
FISH	A labeled single-stranded nucleic acid probe binds to the unknown single-stranded nucleic acid in the sample based on base complementarity, forming a detectable hybrid double-stranded nucleic acid	High sensitivity, high specificity, rapid, Easy to visualize	Photobleaching, autofluorescence, limited to specific sequences	[133]

## 5. Control Strategies

### 5.1. The Control of Bacterial Biofilms in Aquatic Products

Strategies for targeting biofilms can usually be categorized into physical, chemical, and biological methods. Table 5 is a compilation of some specific methods for inhibiting biofilms. The two main new strategies for targeting biofilms are blocking quorum sensing and enzyme-mediated biofilm inhibition.

#### 5.1.1. Physical Methods

Traditional physical methods for biofilm removal include mechanical removal, ultrahigh pressure, and ultrasonic waves. These physical methods have been widely applied to pathogenic bacteria such as *P. aeruginosa*, *S. aureus*, and *E. coli*. Non-thermal plasma treatment is an antimicrobial method that has emerged in recent years. Its main principle is to ionize gases by heating or strong magnetic field, generating a variety of active components (reactive oxygen and nitrogen species, etc.). It can be used to remove or inhibit *P. aeruginosa* biofilm [137]. Electron radiation is a method of disrupting biofilms that has emerged in recent years; it was found that electron beam irradiation reduced the number of viable bacteria in vitro and on the surface of aquatic products, destroying the EPS of the biofilm. The mechanism underlying this phenomenon involves the generation of elevated levels of reactive oxygen species (ROS) upon irradiation, resulting in damage to the bacterial cytoplasmic membrane while preserving the integrity of the cell wall. Furthermore, irradiation results in significant damage to bacterial nucleic acids without altering the primary protein structure. These findings elucidate an intrinsic mechanism by which electron beam irradiation affects Gram-negative bacteria, suggesting its potential efficacy in controlling biofilms formed by spoilage bacteria and pathogenic bacteria on seafood surfaces. Zhou et al. [138] used atmospheric plasma to treat biofilms of spoilage and pathogenic microorganisms on the surface of underwater goldfish, and the results demonstrated that plasma could effectively decrease the biofilm on the surface of fish and hinder the formation of new biofilm. Angarano et al. [139] investigated the inhibition effect of light on the biofilm of the *P. fluorescens* isolated from aquatic products, and the results showed that ultraviolet light and blue light had an effect of removing the biofilm, while green light, yellow light, and red light had no effect on the biofilm, confirming that visible light may be used as a new anti-biofilm strategy. The efficacy of a single physical method for bacterial biofilm removal may be limited; thus, a novel physical method or a combination of methods should be sought.

#### 5.1.2. Chemical Methods

Novel chemical methods mainly utilize natural antimicrobial agents (essential oils (EOs), tea polyphenols, chitosan and other plant and animal extracts). Among these, plant EOs are aromatic volatile compounds extracted from plants, which are widely used in food preservatives [140]. The antimicrobial mechanism and antiviral activity of EOs are mainly attributed to their various phenols and terpenoids. Another new bactericidal technology involves the use of acidic electrolytic water (AEW). AEW is a broad-spectrum, highly efficient, green bactericidal technology that has been widely used in agriculture, medicine, environmental protection, food, and other industries. At present, research into AEW study for the inactivation of plankton microorganisms is relatively mature. Its main bactericidal mechanism is to damage the integrity of cell walls, membranes, and other cellular structures, resulting in rapid leakage of DNA and proteins within the cell. Wang et al. [141] showed that flavonoids extracted from heart grass played a key role in the anti-biofilm activity of *S. putrefaciens* isolated from aquatic products, destroying the biofilm and leading to the death of bacterial cells. Santhakumari et al. [142] investigated the biofilm-inhibitory impact of 2,6-di-tert-butyl-4-methylphenol (DTBMP), an extract of Rhodococcus macrophyllum, on the biofilm of *Vibrio* spp. in aquatic products, showing that DTBMP could prevent initial bacterial adherence and formation of biofilm, interfering with the bacterial adherence to aquatic product. Other studies have shown that benzoate and sorbate have a certain resistance to the biofilm of the *P. fluorescens*; the effect on the plankton bacteria is especially obvious compared with bacteria that have formed mature biofilms [143]. In addition, nanometer-scale benzoic and sorbate showed more significant anti-biofilm properties because their smaller antimicrobial particles easily entered the biofilm and came into direct contact with the bacteria [144].

#### 5.1.3. Biological Methods

Novel biological methods for the removal of bacterial biofilms include phages, lactobacilli bacteriocins, enzymes, and quorum-sensing inhibitors (QSIs). The phage method is a highly specific method that produces depolymerization enzymes to destroy extracellular polysaccharides in biofilms. This is promising for the destruction of bacterial biofilms, but its high specificity is a fatal drawback when dealing with mixed biofilms. A novel phage isolated from *V. parahaemolyticus* acted as an inhibitor of the biofilm of *V. parahaemolyticus* isolated from fish but did not disrupt the existing biofilm [145]. Lactobacillus bacteriocins and enzymes are widely used in the food industry due to their safety [146]. At present, they are used as inhibitors of bacterial biofilm formation. Puga et al. [147] investigated the inhibition of biofilms of common bacteria from seafood, meat, and dairy products using a variety of commercial enzymes and found that DNAase I, streptavidin, and pectinase significantly disrupted the structure of bispecies biofilms, but enzyme-treated bacterial cells survived after extraction. Various bacterial physiological processes, such as production of extracellular proteases and polysaccharides, pigmentation, community motility, and biofilm formation, are regulated by the QS system. Therefore, disrupting the QS system may be a novel way to control bacterial spoilage capacity and production of virulence factors to extend shelf life and increase seafood safety. Controlling the bacterial QS system may be an effective strategy to reduce bacterial spoilage without causing bacterial resistance. QSIs inhibit the quorum sensing phenomenon by inhibiting the production of bacterial water-soluble extracellular polysaccharides and the activity of Ca^2+^-ATPase in aquatic products, which can block the exchange of information between bacteria and inactivate the QS signaling molecules. Since biofilm formation is often dependent on group sensing, QSIs can effectively inhibit biofilm formation. Quorum quenching (QQ) enzymes effectively inhibit bacteria-induced food spoilage by inactivating the QS system and blocking the synthesis of bacterial virulence factors [148]. The quorum sensing inhibitors currently identified in the literature include immobilized penicillin acylase, hesperidin [149], corticotyledonin glycan extract [150], methyl phthalate, resveratrol, and cinnamic aldehyde [151]. Methyl phthalate can be used as a QSI to inhibit the biofilm formation of the turbot spoilage fungus *Aeromonas* spp. Results showed that this inhibitor significantly reduced *Aeromonas*. spp. biofilm formation, motility, protease activity, and the production of AHLs [152]. Cinnamaldehyde also can be used as a QSI for the turbot spoilage bacterium *P. fluorescens*, but instead of destroying the QS signaling molecule AHLs of the bacteria, cinnamaldehyde disrupts the QS system by destroying the bacterium’s LuxR-type proteins and thus, the QS system [153]. Resveratrol inhibited biofilms by disrupting DKPs in induction of the Baltic *Schizosaccharomyces cerevisiae* population and delayed spoilage of large yellow croaker during cold storage [154]. Most of the spoilage bacteria in aquatic products rely on QS to form biofilms, and QSIs inhibit biofilm formation by disrupting the QS system [2]. Therefore, the research on new green QSIs as preservatives for aquatic products is gradually increasing.

**Table 5:** Methods of inhibiting biofilms.

Method	Categories	Mechanism of Inhibition	Reference
Non-thermal plasma	Physical method	Ionization of gases by heat or a strong magnetic field generates a variety of active ingredients (superoxide, photons, etc.) that work in synergy to remove the bacterial biofilm	[137]
Electron beam radiation		Elevation of ROS levels by electron beam irradiation leading to damage to the bacterial cytoplasmic membrane and damage to bacterial nucleic acids	[138]
Phage method	Biological method	Production of depolymerases to disrupt extracellular polysaccharides in biofilms	[145]
Methyl phthalate		Reduces *P. aeruginosa* biofilm formation, motility, protease activity, and production of AHLs (high serine lactones)	[152]
Cinnamaldehyde		Disrupts the QS system by destroying the bacteriophage’s LuxR-type proteins	[153]
Resveratrol		Inhibition of biofilms by disruption of diketopiperazine analogues (DKPs) in population sensing	[154]
EOs	Chemical method	Flavonoids contained in EOs can disrupt biofilms and cause bacterial cell death	[140]
2,6-Di-tert-butyl-4-methylphenol (DTBMP)		Prevents initial bacterial adhesion and biofilm formation and interferes with bacterial adhesion to aquatic products	[142]

### 5.2. Inhibiting Bacterial Growth

#### 5.2.1. Control Oxygen Content

Oxygen serves as a critical electron acceptor in the metabolic processes of obligate aerobic bacteria and is usually a privileged acceptor for parthenogenetic anaerobes. Reduced levels of oxygen in the environment can influence bacterial metabolism and reduce growth rates. On the contrary, too high an oxygen concentration can have an adverse effect on bacterial growth, as oxygen promotes oxidative reactions. Optimal growth conditions for most aerobic species align with atmospheric oxygen levels, with inhibition occurring only under significant deviations from these levels. *Pseudomonas* spp. are aerobic microorganisms that can grow only in the presence of molecular oxygen. If the dissolved oxygen concentration is too low, the growth of the bacteria is slow and the enzyme-producing capacity of the cells is also weakened [155]. Therefore, the growth of *Pseudomonas* spp. can be inhibited by reducing oxygen; for example, the number of *Pseudomonas* bacteria was significantly reduced in fish stored after being vacuumed and packaged with carbon monoxide. Oxygen scavengers have been extensively studied in active packaging, and the use of oxygen scavengers can be very effective in reducing the level of oxygen residue in packaging to less than 100 ppm, effectively controlling fat oxidation and inhibiting microbial growth, thus preserving the product quality and prolonging storage life [156].

#### 5.2.2. Use of Antimicrobials

**Figure 8:**
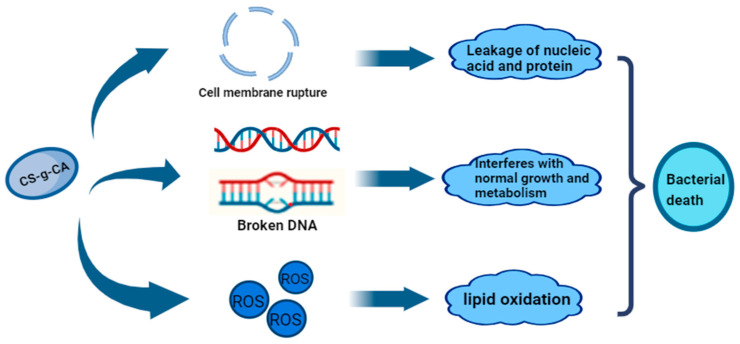
Antimicrobial mechanism of CS-g-CA against *P. fluorescein*. The wavy lines in red and blue usually represent the principle of base pairing. In this model, the red wavy line may represent the pairing of adenine (A) and thymine (T), while the blue wavy line may represent the pairing of cytosine (C) and guanine (G).

Antimicrobials can be used to inhibit bacterial growth. Therefore, the search for effective antimicrobial agents continues to receive increasing attention. Recently, there have also been more studies on the development of antimicrobial agents for *P. aeruginosa*, such as chitosan caffeic acid grafts, representing a new type of anti-*P. aeruginosa* agent. tLan et al. [157] found that this grafting method was able to disrupt the microstructure of *P. aeruginosa*, leading to cell membrane rupture, inracellular material leakage, and accumulation of reactive oxygen species. Excess reactive oxygen species can cause lipid peroxidation, destroy the membrane structure of *P. fluorescence*, and produce lipid peroxides. In addition, the DNA of *P. fluorescens* was destroyed by the graft, further resulting in bacterial death, and the grafting also affected the motility of *P. fluorescens*. Finally, the cells of the bacteria were unable to grow and multiply properly, leading to lysis and death. The antibacterial mechanism of CS-g-CA against *P. fluorescens* is shown in Figure 8. Studies have shown [158] that terpinen-4-ol can effectively damage the cell wall of *P. fluorescens*, enhancing the permeability of the cell membrane, leading to the extravasation of intracellular ions and irreversible damage to the cell membrane, in addition to the leakage of large molecules of DNA, reducing the amount of intracellular proteins, blocking the expression of proteins and the synthesis of ATPase, and resulting in cell apoptosis. Alkyl gallate also has an inhibitory effect on *P. fluorescens* [159]. Octyl gallate (GAC8) quickly penetrates the lipid bilayer part of the membrane to disorganize the membrane and further impedes the growth of *P. fluorescens* by disturbing the tricarboxylic acid cycle, which is associated with energy supply, as well as amino acid metabolism, which is associated with the cell membrane, inhibiting oxygen consumption and interfering with respiratory chain. In addition, GAC8 can disrupt the fatty acid composition of the cell membranes, making bacteria more sensitive to antimicrobial agents, and can cause structural damage to the bacterial membranes.

#### 5.2.3. Preservation Coating Technology

Coating technologies offer promising avenues for combating biofilm formation within various marine infrastructures crucial to the shellfish industry. For example, surface biofilm formation facilitated by *Pseudomonas* spp. was effectively inhibited through the application of a biodegradable, wax-based, and nontoxic coating substance [160]. D-tryptophan/polylactic acid coating material and sodium chloride composed of preservative and freshness-coating film [161] can regulate antimicrobial timeliness with long-term effects. Novel types of nano-antimicrobial composite anticorrosion film with recycling functions [162,163] can greatly extend the preservation time of aquatic products. Nanoparticles (NPs) loaded with proanthocyanidins (PCs) obtained by cross-linking chitosan (CH) and chondroitin sulfate (CS) were added to protective film to provide stronger antioxidant, antimicrobial, and oxygen-blocking effects [164].

### 5.3. Anti-Bacterial Packaging Materials

Food packaging materials play a pivotal role in controlling food quality and ensuring food safety. With growing concern for the environment, sustainable and environmentally friendly natural biopolymer films have been developed as alternative packaging materials to traditional synthetic plastics. Low-density polyethylene, polydichloroethylene, polyvinyl chloride, starch, pectin, carrageenan, chitosan, gelatin, and so on, have great potential for preparing food packaging films to maintain or improve food quality. For example, packaging based on chitosan/polyvinyl alcohol and ginger essential oil-loaded bacterial cellulose was applied for fresh-keeping packaging of bass; the experimental results showed that the antibacterial activity of the film against *S. aureus*, *E. coli*, and *P. fluorescens* was significantly improved [165]. In addition, antibacterial agents have also been applied to plastic foams used as packaging materials for the preparation of packaging suitable for vegetables, fruits, and aquatic products. The foam material is made of high-strength polystyrene, polyethylene, or polypropylene and can also be used in incubators for cold chain transport of aquatic products [166].

## 6. Conclusions and Future Prospects

The contamination characteristics of *Pseudomonas* spp. in aquatic products and its influence on food quality are systematically discussed in this paper. *Pseudomonas* psychrophila is known for its adaptability to growth and biofilm formation at low temperatures, and it can produce a variety of volatile compounds by decomposing proteins and amino acids in aquatic products, leading to corruption of these products and associated safety risks. This article reviews a variety of detection methods and control strategies, including physical, chemical, and biological methods, with particular emphasis on biofilm-specific inhibition techniques such as inhibition of quorum sensing and the application of natural antimicrobials. These studies provide a theoretical basis and practical guidance for the preservation and processing of aquatic products.

Future studies should focus on the development of novel natural anti-biofilm products to deal with the increasing problem of *Pseudomonas* psychrophila contamination in the aquatic product industry. In addition, it is of great significance to explore the application of nanotechnology, probiotics, and biosurfactants in controlling *Pseudomonas* psychrophila. As consumption of aquatic products increases globally, ensuring the safety and quality of aquatic products will be a key public health challenge. Therefore, the comprehensive use of existing technologies and emerging methods to establish a multi-level prevention and control system will help improve the effective preservation of aquatic products and ensure the health and safety of consumers.

## Figures and Tables

**Table 1:** Species and characteristics of aquatic products.

Species	Compounds	Properties	Reference
Fish	Proteins, omega-3 polyunsaturated fatty acids, vitamin D and B vitamins, eicosapentaenoic acid (EPA), docosahexaenoic acid (DHA)	Antioxidant, helps cardiovascular health, improves brain function, reduces inflammation, helps with bone health	[7]
Shrimp	Proteins, minerals, particularly selenium, astaxanthin	Antioxidant, antihypertensive, antibacterial	[8]
Crab	Bioactive peptides, minerals, chitin	Antiviral, enhances immunity, promotes blood circulation	[9]
Bivalves	Oyster peptide, alginate, vitamin B12, squalene, polysaccharide, sulfides, organic acids	Antioxidant, anti-fatigue, enhances immunity, maintains nervous system and red blood cell health	[10]

**Table 2:** Species of *Pseudomonas* spp. in different aquatic products (partially displayed).

Fish Species	Species (And Strains)	Spoilage Characteristics	References
Sturgeon (*Acipenser baerii*)	*P* *. fluorescens*	Fat oxidation	[26,27]
Grass carp (*Ctenopharyngodon idella*)	*P. malodorata*	Degraded amino acid	[23]
Turbot (*Scophthalmus maximus*)	*P. fluorescens* PF08	Protease production, biofilm formation, and sulfur and amine metabolism	[21,28]
Salmon (*Salmo salar*)	*P. aeruginosa*	Lipid oxidation, biofilm formation	[29]
Tilapia (*Oreochromis niloticus)*	*P. fragi* BBa3	Protein hydrolysis and oxidation	[30]
Grouper (*Epinephelus fuscoguttatus*)	*P. fluorescens*, *P. aeruginosa*	Formation of undesirable odors and flavors	[31]
Large yellow croaker (*Larimichthys crocea*)	*P. plecoglossicida*, *P. fluorescens*	Discoloration and ulceration of fish	[32]

## Data Availability

The datasets generated during the current study are not publicly available because the data form part of an ongoing study, but data are available from the corresponding author on reasonable request.

## Abbreviations

EPA	Eicosapentaenoic acid
DHA	Docosahexaenoic acid
CSPs	Cold shock proteins
Pro	Proline
Gly	Glycine
3-D	Three-dimensional
IBPs	Ice binding proteins
AFPs	Antifreeze proteins
INPs	Ice nucleation proteins
TH	Thermal hysteresis
EPS	Extracellular polymers
C-di-GMP	Cyclic di-guanosine monophosphate
DGC	Diguanylate cyclase
PDE	Phosphodiesterases
GGDEF	Gly-Gly-Asp-Glu-Phe
QS	Quorum sensing
AHLs	N-acyl-homoserine lactones
AIPs	Autoinducing peptides
AI-2	Autoinducer-2
DKPs	Diketopiperazines
TCS	Two-component control system
HK	Histidine kinase
RP	Regulatory protein
TVC	Total viable counts
VOCs	Volatile organic compounds
BAs	Biogenic amines
TMA	Trimethylamines
HS-SPME	Headspace solid-phase microextraction
GC-MS	Chromatography-mass spectrometry
ADC	Arginine decarboxylases
HPLC	High-performance liquid chromatography
GC	Gas chromatography
CZE	Capillary zone electrophoresis
IEC	Ion-exchange chromatography
DMA	Dimethylamine
FA	Formaldehyde
IMS	Ion mobility spectrometry
TVB-N	Total Volatile Base Nitrogen
SSOs	Specific spoilage organisms
TBA	Thiobarbituric Acid
ATP	Adenosine triphosphate
ADP	Adenosine diphosphate
AMP	Adenosine monophosphate
IMP	Inosine monophosphate
HxR	Inosine
Hx	Hypoxanthine
RPA	Polymerase amplification
PCR	Polymerase chain reaction
LAMP	Loop-mediated isothermal amplification
NGS	Next-generation sequencing
FISH	Fluorescence in situ hybridization
MLST	Multilocus sequence typing
ROS	Reactive oxygen species
EO	Essential oils
AEW	Acidic electrolytic water
DTBMP	2,6-di-tert-butyl-4-methylphenol
QSIs	Quorum-sensing inhibitors
QQ	Quorum quenching
GAC8	Octyl gallate
NPs	Nanoparticles
PCs	Proanthocyanidins
CH	Chitosan
CS	Chondroitin sulfate
